# *TXM-Pal*: a companion software for advanced data processing in spectroscopic X-ray microscopy

**DOI:** 10.1107/S1600577525002036

**Published:** 2025-04-02

**Authors:** Sugeun Jo, Sangwoo Kim, Jun Lim

**Affiliations:** a Pohang Accelerator Laboratory Pohang University of Science and Technology (POSTECH) 80 Jigok-ro 127 beon-gil, Nam-gu Pohang-si Gyeongsangbuk-do37637 Republic of Korea; Tohoku University, Japan

**Keywords:** transmission X-ray microscopy (TXM), X-ray image analysis, X-ray absorption near-edge structure (XANES) imaging, X-ray spectroscopy, lithium ion batteries

## Abstract

Transmission X-ray microscopy (TXM) is a powerful and non-destructive tool for interpreting the chemical states of a wide range of materials. It utilizes X-ray absorption near-edge structure (XANES) for the chemically sensitive mapping of specific elements, making it particularly effective for studying heterogeneous systems. However, specialized software is required for XANES imaging analyses to precisely align and correct the image drift, ensuring accurate XANES fitting. To address this issue, we developed *TXM-Pal*, a Python-based software optimized for XANES imaging data analysis at the 7C-XNI beamline in Pohang Light Source II. This software includes a user-friendly graphical user interface that facilitates XANES analysis, allowing images to be processed within a few minutes. The *Rust*-based implementation in *TXM-Pal* accelerates data analysis by tens of times compared with programs of pure Python. Using *TXM-Pal*, we procured detailed insights into the XANES analytical workflow. To demonstrate the efficacy of the proposed software, we mapped the Ni oxidation states within lithium-ion battery cathodes and thereby revealed their heterogeneity. *TXM-Pal* will be continuously updated to enhance XANES imaging analysis for users at spectroscopic X-ray microscope beamlines.

## Abstract 2

Advanced software for X-ray spectroscopy imaging has been developed, enabling precise and rapid XANES imaging analysis. Its effectiveness has been demonstrated by mapping the Ni oxidation states in lithium-ion battery cathodes, thereby revealing their heterogeneity.

## 1. Introduction

The short wavelengths of synchrotron-based hard X-rays enable high spatial resolution and deep-matter penetration in full-field transmission X-ray microscopy (TXM), making them highly suitable for non-destructive analyses of advanced materials (Meirer *et al.*, 2011 ▸; Yuan *et al.*, 2012 ▸; Li *et al.*, 2018 ▸; Wang *et al.*, 2020 ▸). Notably, TXM provides nano-computed tomography (nano-CT) imaging, revealing sub-particle-level defects (Li *et al.*, 2022 ▸; Tao *et al.*, 2023 ▸; De Andrade *et al.*, 2021 ▸; Zeng *et al.*, 2022 ▸; Jo *et al.*, 2024*a* ▸) with high 3D morphological detail at a spatial resolution of approximately 30 nm (Yu *et al.*, 2020 ▸; Tang *et al.*, 2021 ▸). By utilizing X-ray absorption near-edge structure (XANES), which employs the dramatic change in the X-ray absorption coefficient at the absorption edge, it is possible to visualize the chemically sensitive mapping of each element within a sample (Gent *et al.*, 2016 ▸; Yu *et al.*, 2015 ▸; Mao *et al.*, 2019 ▸; Jo *et al.*, 2023 ▸; Wang *et al.*, 2023 ▸). The integration of CT and TXM–XANES allows for detailed visualization of both the morphologies and the specific elemental chemical states of materials in 3D (Liu *et al.*, 2019 ▸; Huang *et al.*, 2024 ▸).

Typical bulk XAFS analyses, which do not require image alignment, rely on established software such as *Athena* (Ravel & Newville, 2005 ▸). In contrast, XANES imaging analyses require precise alignment to correct for image drifts that occur during data acquisition. These drifts are typically incurred by factors such as X-ray beam instability, device thermal shifts and sample movements on the scale of tens of nanometres during energy scans (Zhang *et al.*, 2021 ▸; Welborn *et al.*, 2024 ▸). Therefore, XANES imaging analyses require specialized tools designed for spectroscopic X-ray microscopy. For instance, the TXM team at the Stanford Synchrotron Radiation Light Source (SSRL) developed the *TXM-Wizard* software (Meirer *et al.*, 2011 ▸; Liu *et al.*, 2012 ▸), whereas the team at the National Synchrotron Light Source (NSLS) II developed *TXM-Sandbox* and *PyXAS* (Xiao *et al.*, 2022 ▸; Ge & Lee, 2020 ▸). For many years, *aXis2000* (Hitchcock, 2023 ▸) and *MANTiS* (Lerotic *et al.*, 2014 ▸) have been widely used in the field of scanning transmission X-ray microscopy. These tools facilitate the high-throughput visualization of XANES images and ensure robust imaging alignment.

XANES nano-imaging was previously installed on the 7C-XNI beamline at Pohang Light Source (PLS) II, which established a zone-plate-based full-field TXM (Park *et al.*, 2020 ▸). X-rays, generated by an in-vacuum undulator and monochromated by a liquid-nitro­gen-cooled double-crystal monochromator (DCM), were collimated using a rhodium-coated horizontal focusing plane mirror (HFM). A beam-shaping condenser zone plate with pitches of 152, 295, 270 and 437 nm (from inner to outer), and a 500 µm diameter produces flat-top illumination on the sample, matching the field of view (FOV) in the on-axis illumination system. Behind the sample, the objective Fresnel zone plate is deployed, featuring a 30 nm outermost zone width and 300 µm diameter. The X-ray image is then converted to the visible range by a 20 µm-thick scintillation crystal (GGAG), magnified by a 20× optical objective lens and captured by an sCMOS camera with 2048 × 2048 sensors, each having a 6.5 µm pixel size. The typical FOV is approximately 51 µm, and the effective pixel size, with two bins, is approximately 50 nm at approximately 8.33 keV (Ni *K*-edge). In the XANES imaging experiment, projection and background images were captured in an energy series from which a single XANES spectrum was extracted from a single pixel. Despite these capabilities, the 7C-XNI beamline currently lacks optimized XANES analysis software.

In this context, we developed *TXM-Pal*, a Python-based software program designed to facilitate fast and efficient XANES imaging data analyses at the 7C-XNI beamline of PLS-II. *TXM-Pal* features a user-friendly graphical user interface (GUI) that simplifies the analytical process, as well as precise alignment methods. We demonstrate the functionality of *TXM-Pal* using various examples, with the *Rust*-based achieving data analysis speeds up to tens of times faster than reached purely with Python. Furthermore, *TXM-Pal* provided detailed insights into each step of workflow, which encompassed lithium-ion battery cathodes and displayed heterogeneity within cathode particles by mapping the Ni oxidation states. *TXM-Pal* will continue to be updated to assist users in XANES imaging analyses at the 7C-XNI beamline.

## 2. XANES analytical work process

**Figure 1:**
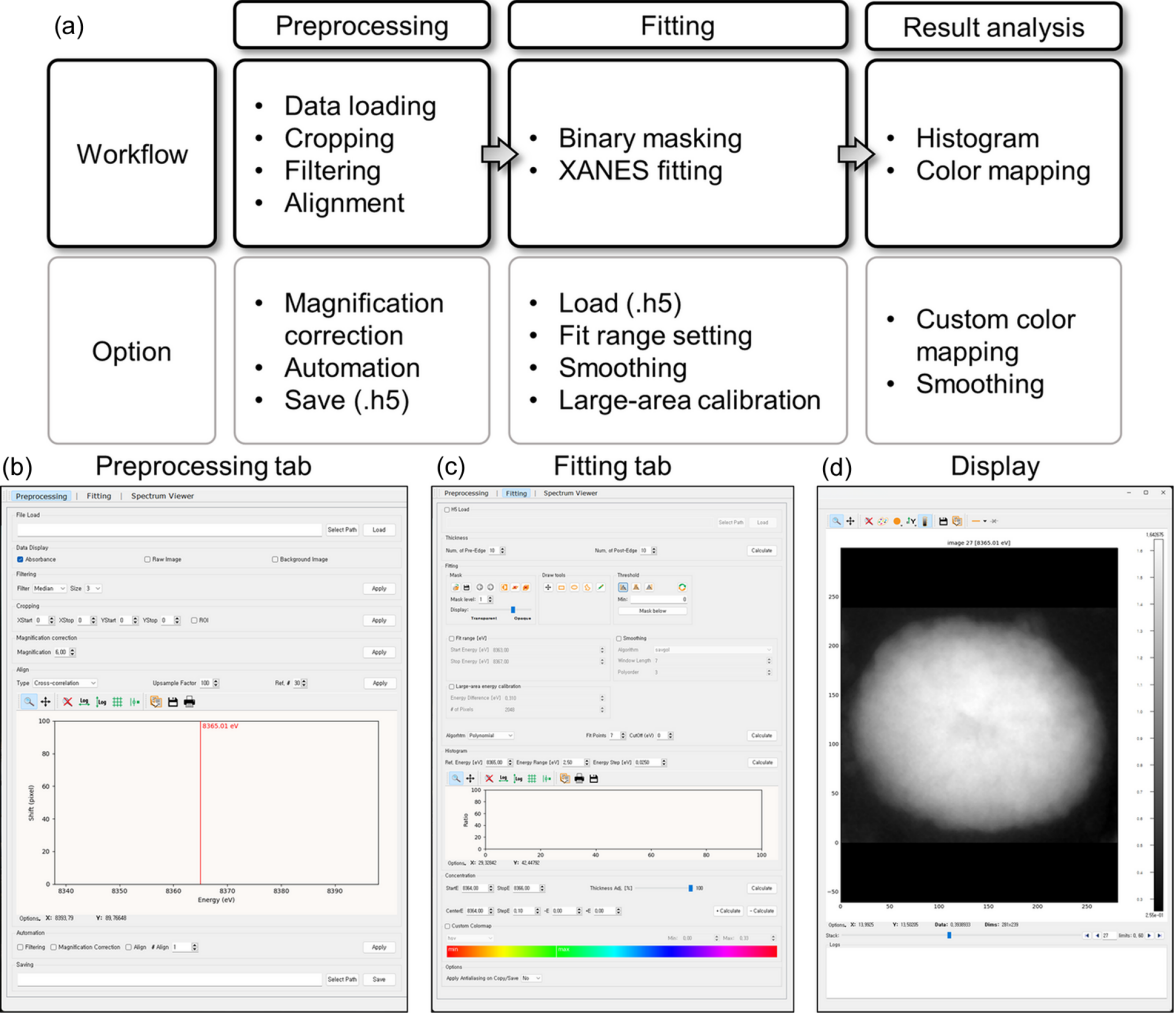
Overview of XANES analysis on *TXM-Pal*. (*a*) Workflow of XANES analysis using *TXM-Pal*. (*b*)–(*d*) Screenshots of *TXM-Pal* GUI. (*b*) Preprocessing tab: includes data loading, cropping, filtering, alignment, optional magnification correction, automation and saving to .h5 file. (*c*) Fitting tab: includes binary masking, XANES fitting, histogram drawing and color mapping, with options to load a .h5 file and apply fit range setting, spectrum smoothing, large-area calibration, custom color mapping and image smoothing. (*d*) Display screen: located on the right side of the fitting/preprocessing tab screen.

A *TXM-Pal*-facilitated XANES analysis was performed using the following steps: (1) data loading, (2) cropping, (3) filtering and magnification correction, (4) alignment, (5) binary masking, (6) XANES fitting, and (7) color mapping and histogram. Subsequently, the spectrum viewer was used to verify the reliability of results from specific pixels or regions of interest (ROIs). The overall analytical workflow is illustrated in Fig. 1 ▸(*a*), with Figs. 1 ▸(*b*), 1 ▸(*c*) and 1 ▸(*d*) showing detailed screenshots of the *TXM-Pal* GUI. Fig. 1 ▸(*b*) details the preprocessing tab, which covers steps (1)–(4) and includes features for magnification correction, automation and the option to save the processed data in a .h5 file. Fig. 1 ▸(*c*) shows the fitting tab, which encompasses steps (5)–(7) and allows the users to load an existing .h5 file. Fig. 1 ▸(*d*) shows a screen located on the right side of the Fitting/Preprocessing tab. Further details on each phase of the workflow are present in the subsequent sections.

### 2.1. Data loading and cropping

**Figure 2:**
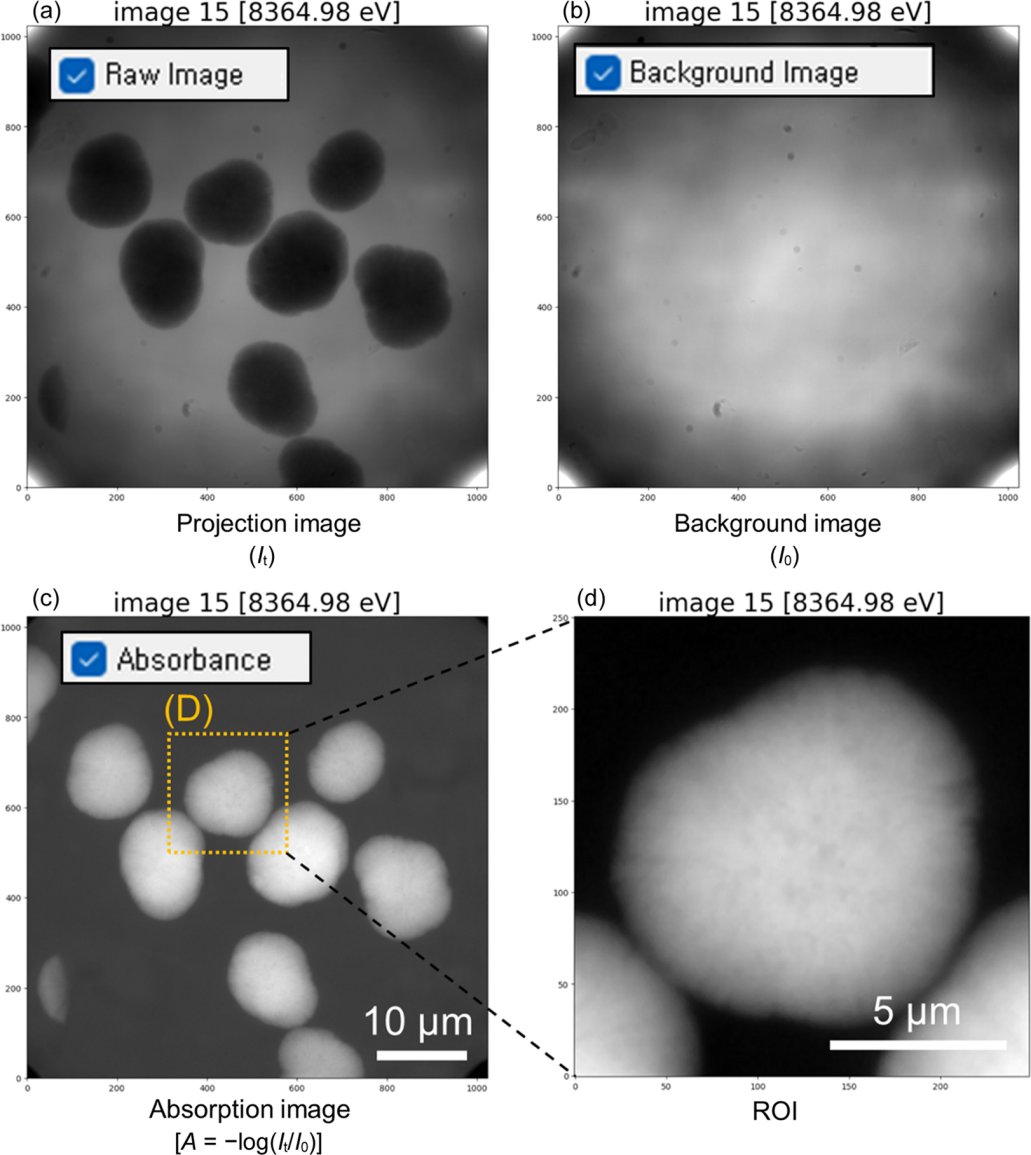
Display of loaded images and ROI definition of Ni-rich layered oxide cathode particles. (*a*) Projection image (*I*_t_), (*b*) background image (*I*_o_) and (*c*) absorption image [−ln(*T*), where *T* = *I*_t_/*I*_o_]. (*d*) Absorption image of ROI-defined cathode particle.

TXM–XANES imaging involves stacks of projection (*I*_t_) and background (*I*_o_) images at each energy. In this study, we used secondary particles of LiNi_0.8_Co_0.15_Al_0.05_O_2_ cathodes as the TXM–XANES imaging sample. XANES scans were performed with 1 eV step sizes around the Ni *K*-edge, covering the energy range 8350–8380 eV. At each energy point, projection (*I*_t_) and background (*I*_o_) images were acquired, ensuring a one-to-one correspondence between *I*_o_ and *I*_t_ files. The FOV was approximately 51 µm, with an effective pixel size of ∼50 nm. Through the preprocessing tab, users can input a folder ending with ‘_proj’ by selecting ‘Select Path’ under the ‘File Load’ section, thereby loading TIF files containing *I*_t_. Subsequently, the software automatically retrieves the corresponding *I*_o_ TIF files from a folder with the same name but ending with ‘_back’. Conversely, if a folder ending in ‘_back’ is selected, the corresponding ‘_proj’ folder is loaded automatically. Furthermore, if multiple images are captured at the same energy to improve image data quality, they are automatically averaged into a single image before being loaded. The TIFF file loading process typically completes within 10 s, with the ‘Logs’ section at the bottom right of the screen displaying each step and its elapsed time, such as ‘loading images…done (6.58 s)’ and ‘back_images…done (6.53 s)’. Once the data are loaded, users can visualize different types of image stacks through the ‘Data Display’ section: projection images (*I*_t_) [Fig. 2 ▸(*a*)], background images (*I*_o_) [Fig. 2 ▸(*b*)] and absorbance images [−ln(*T*), where *T* =*I*_t_/*I*_o_] [Fig. 2 ▸(*c*)]. The ‘Stack:’ shown below the display screen allows users to scroll through energies to quickly check for any issues with the image data collected at the beginning of the analytical process.

Prior to main image analysis, *TXM-Pal* allows for the specification of an ROI [Fig. 2 ▸(*d*)]. Defining an ROI, particularly around a particle of interest, not only ensures a more reliable alignment by focusing on the particle, but also significantly reduces computation time for subsequent analyses, thereby facilitating the overall process.

### 2.2. Filtering, magnification correction, alignment and automation

In X-ray image analysis, the initial preprocessing typically involves noise filtering to enhance the image quality. *TXM-Pal* employs a median filtering process that is widely used in conventional TXM image processing (Liu *et al.*, 2012 ▸), where the median filter substitutes the value of each pixel with the median of its neighboring pixels. Using a 3 × 3 pixel window, this method effectively preserves the image edges while minimizing blurring and removing point noise.

Following noise filtering, magnification correction is necessary to compensate for zone plate movement along the beam axis. In the TXM–XANES system, the sample and detector remain stationary, while the zone plate shifts with energy changes due to its energy-dependent focal length. As a result, both the sample-to-ZP distance (*a*) and the ZP-to-detector distance (*b*) vary, altering the magnification factor (*M* = *b*/*a*). To determine the corrected magnification, we first calculate a reference *M* value at a specific energy using the lens equation (Liu *et al.*, 2012 ▸). At the 7C-XNI beamline, where the system is configured for Ni *K*-edge (8333 eV), *M* = 19.457. Similarly, for the Co *K*-edge (7709 eV) and Mn *K*-edge (6539 eV), *M* = 21.202 and 25.370, respectively. Once this reference *M* value is established, the magnification at any other energy can be derived accordingly. Since *TXM-Pal* records the energy value for each image, it can automatically compute the corresponding *M* values for all energy slices. These computed relative magnification factors are normalized to the initial energy and applied to the final image correction via an affine transformation, specifically a scale transformation. For instance, when scanning from 8350 to 8380 eV, the corresponding *M* values are 19.413 and 19.336, respectively. This indicates that the image at 8380 eV is scaled down by a factor of 19.336/19.413 = 0.9960 relative to the image at 8350 eV. *TXM-Pal* applies this correction automatically, ensuring consistent magnification across all images and compensating for shifts in the zone plate position. This correction is unnecessary in projection X-ray microscopy without zone-plate optics.

**Figure 3:**
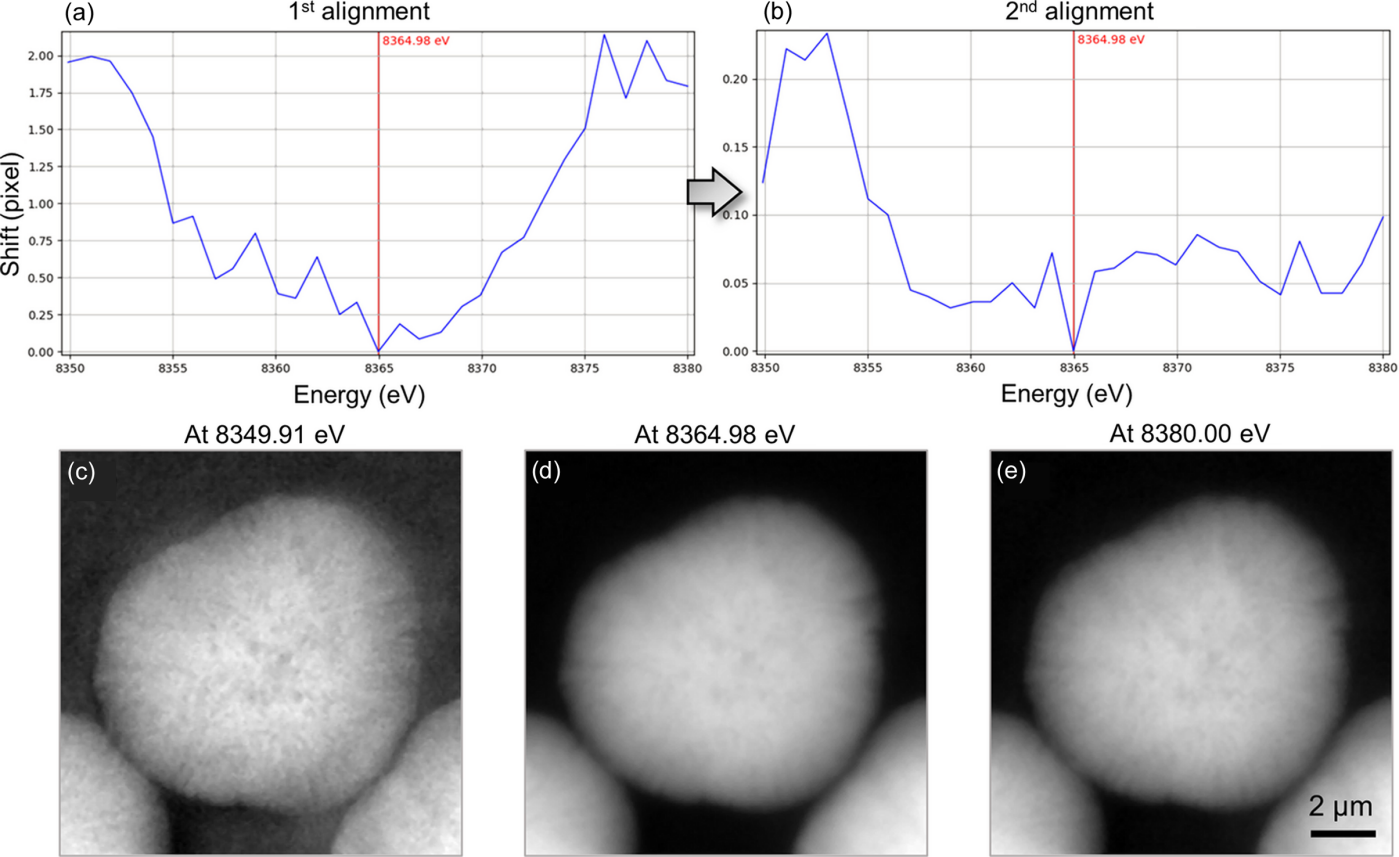
Example of alignment results. Screenshots of image alignment results in *TXM-Pal*, indicating the pixelwise shifts at different energies during the (*a*) first and (*b*) second alignment iterations. Transmission images at (*c*) 8349.91, (*d*) 8364.98 and (*e*) 8380.00 eV after magnification correction and second alignment.

Alignment is then performed to correct for image drifts that occur during data acquisition, which typically stem from X-ray beam instability, device thermal shifts and sample movements on the scale of tens of nanometres during energy scans. *TXM-Pal* offers two image registration algorithms – cross-correlation and *StackReg* (Thévenaz *et al.*, 1998 ▸) – for aligning the XANES images stack. When a user selects an image from the stack as a reference, the software aligns the remaining images accordingly, displaying the number of shifted pixels at different energies. These displacements are initially measured in both the *x* and the *y* directions. To provide a concise quantitative assessment, we compute a single scalar displacement value for each image by taking the square root of the sum of the squared *x* and *y* shifts. Alignment is typically adjusted to the subpixel level to enhance accuracy. For instance, using 8365.0 eV as a reference image and applying the cross-correlation method, the drifts were effectively corrected, demonstrating that the images shifted by 0–100 nm relative to the reference image [Fig. 3 ▸(*a*)]. Further alignment revealed that the shifted pixels were tuned to the sub-pixel range 0–10 nm relative to the reference image [Fig. 3 ▸(*b*)]. Using the ‘Stack:’ scroll confirmed that no drifts were observed with energy changes following the two alignments [Figs. 3 ▸(*c*), 3 ▸(*d*) and 3 ▸(*e*)]. Although the alignment iteration is not a constant, it typically ranges between 1 and 3 depending on the sample.

To analyze efficiency, *TXM-Pal* incorporates a function that automatically applies filtering, magnification correction and alignment, with users able to adjust the number of alignment iterations. This automation is particularly useful for handling large time-intensive datasets, saving users 2–3 min which can then be used to conduct further beamline operations or discuss previous experimental results with coworkers. The intermediate results are saved in .h5 format until the alignment stage, enabling users to reload these files in future sessions without repeating the initial processes.

### 2.3. Binary masking

**Figure 4:**
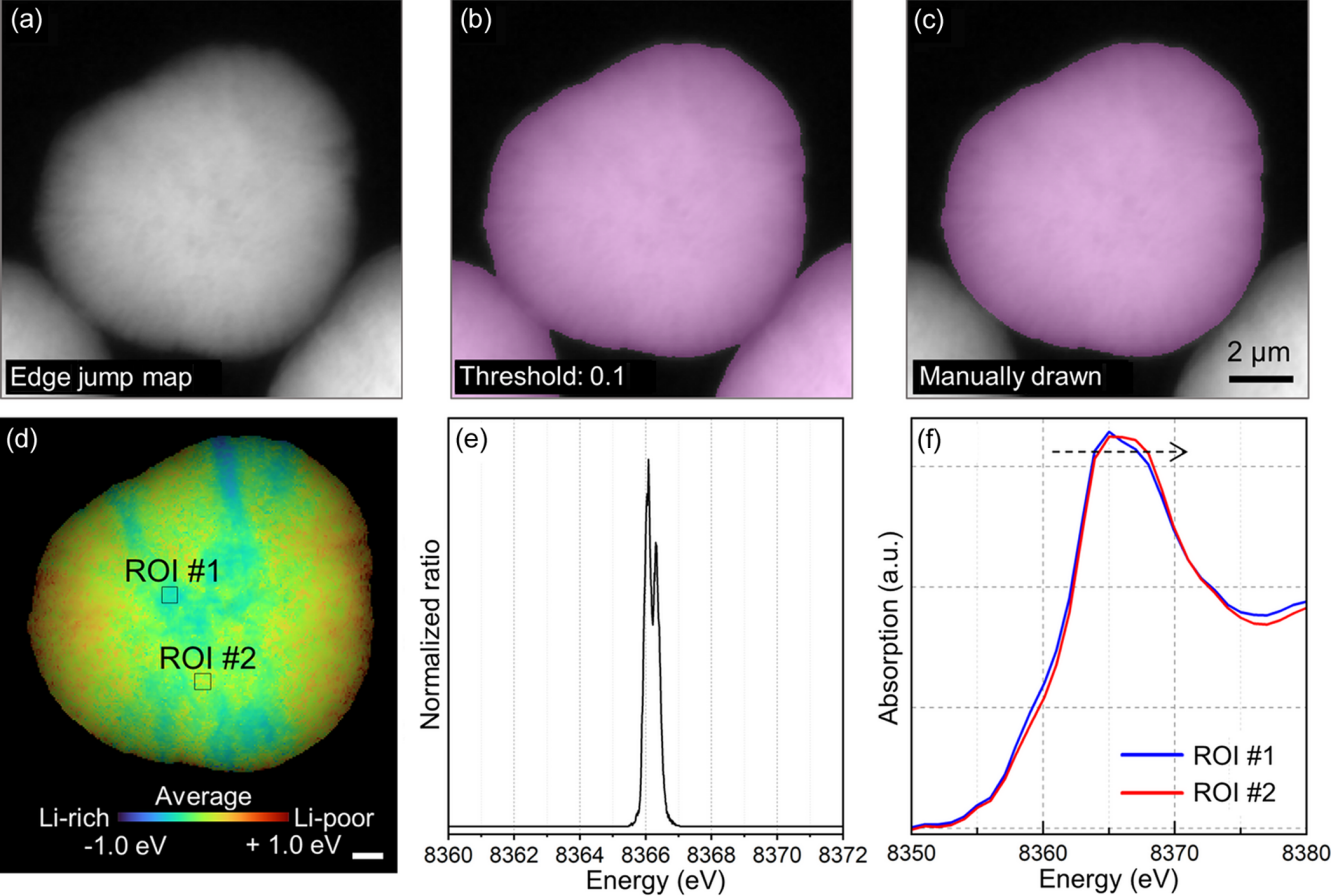
Examples of binary masking, XANES fitting and color mapping. (*a*) Edge-jump image of an Ni-rich LiNi_*x*_Co_*y*_Al_*z*_O_2_ cathode particle. Masks generated from the edge jump image, (*b*) with a threshold set at 0.1 and (*c*) manually drawn. (*d*) Color mapping of an Ni-rich LiNi_*x*_Co_*y*_Mn_*z*_O_2_ cathode particle, with Li-rich and Li-poor regions depicted in navy and red, respectively. Scale bar set to 1 µm. (*e*) Normalized histogram for the peak position distribution of the particle. (*f*) Comparison of averaged XANES spectra in ROIs between ROI-1 and ROI-2 shown in (*d*).

The binary masking process is conducted in the ‘Fitting’ tab. Users can use the aligned images from the previous tab or load previously saved images in .h5 format using the ‘H5 Load’ option. Even after defining an ROI, multiple irrelevant particles or empty spaces may remain. To calculate accurate quantitative information – for instance, the mean peak position energy or standard deviation for a specific particle – we apply a binary mask that isolates only the ROIs. A binary mask is generated by applying the threshold of the edge jump map, which is derived from differences between the average pre-edge and post-edge values for each pixel. To create this edge-jump map, users can specify how many images at the beginning and end of the stack to treat as the pre-edge and post-edge regions, respectively. Since the edge jump correlates with the local penetration thickness of a specific element, the binary mask can be obtained by adjusting the edge-jump threshold [Fig. 4 ▸(*a*)]. By adjusting the threshold, a binary mask is created and the transparency of the magenta mask can be adjusted in the ‘Mask’ menu [Fig. 4 ▸(*b*)]. Additionally, masks can be manually selected using draw tools, which can be used to exclude specific parts of the sample [Fig. 4 ▸(*c*)].

### 2.4. XANES fitting

In *TXM-Pal*, the peak position fitting of XANES is utilized to map the chemical state. To define the XANES peak position, the maximum point in the XANES spectra is initially identified for each pixel. Around this maximum point, the set fit points are fitted using either a second-order polynomial or a Gaussian function, and the vertex of the fitted curve is defined as the peak position of each pixel. Users can adjust the ‘Fit points’ variable to manipulate the energy range required to fit points. For example, if an energy scan is performed in 1 eV steps, users can fit within an energy range of ±3 eV around the peak maximum by setting the ‘Fit Points’ variable to 7. Typically, the energy range is adjusted to within ranges of ±2 to ±3 eV. We note that using the edge position in XANES is a relatively precise method for tracking changes in oxidation state in transition metals, particularly those exhibiting significant variations in the XANES region. However, most XANES imaging experiments conducted at the 7C-XNI beamline focus on lithium-ion battery cathodes, specifically Ni-rich layered oxides, where the Ni *K*-edge peak position exhibits an approximately linear correlation with the oxidation state (Jo *et al.*, 2023 ▸; Tsai *et al.*, 2005 ▸; Gent *et al.*, 2016 ▸). Future updates to our software will include options for this edge fitting, as outlined in Section 4. Until these features are implemented, we advise users that peak fitting may not always yield the most accurate determination of oxidation state for all samples.

Before initiating the fitting calculation, users can adjust several optional settings to enhance analytical accuracy: (i) fit range: this setting confines the fitting to a specified range; (ii) smoothing: this feature, which smooths the spectrum of each pixel before fitting, is recommended only for cases involving highly noisy data, such as when samples inadequately absorb X-rays; (iii) large-area energy calibration: exclusive to the projection X-ray microscope system at the 7C-XNI beamline of PLS II, this setting compensates for the energy gradient caused by the diverging beam in the vertical direction. For instance, at the Ni *K*-edge, the typical calibration value is approximately 0.31 eV at 2048 pixels. This calibration is not necessary for a typical TXM setup.

### 2.5. Color mapping, histogram and spectrum viewer

The fitted peak positions of samples were visualized using various color maps. By default, we use the hue-saturation-value (HSV) color space, where the hue ranges from 0.0 (red) to 0.33 (green). Here, 0.0 and 0.33 refer to normalized hue values in HSV, corresponding to ∼0° (red) and ∼120° (green). The difference in absorption between the pre- and post-edge regions determines the brightness of each pixel. Users can customize the color scheme using options such as turbo, jet or rainbow. For instance, in the ‘turbo’ color scheme, colors are mapped from navy (0) to red (1). In this scheme, the Li-rich regions of the LiNi_*x*_Co_*y*_Mn_*z*_O_2_ cathode are depicted in navy while the Li-poor regions are shown in red, as shown in Fig. 4 ▸(*d*). For *in situ* experiments, setting StartE and StopE to the peak positions of the fully discharged and charged states, respectively, illustrates the state of the charge on the color map. Users can save these images in PNG format. Applying Gaussian blurring as an anti-aliasing method helps to eliminate point artifacts.

The peak positions assigned to each pixel can be displayed as normalized ratios in a histogram [Fig. 4 ▸(*e*)], which users can save as an ASCII file using the save button or quickly copy with the copy button.

Subsequently, the spectrum viewer can be used to verify the reliability of the fitting results from specific pixels or ROIs. Users can examine the spectrum by specifying an ROI on a color-mapped particle. For instance, selecting the navy and red areas as ROI-1 and ROI-2, respectively, as depicted in Fig. 4 ▸(*d*), shows that the spectrum of ROI-2 shifted further to the right than that of ROI-1 [Fig. 4 ▸(*f*)]. Although the energy shift may not be immediately apparent, even small differences in oxidation state can significantly impact battery performance. Recent studies have reported that anisotropic strain during cycling likely induces such subtle variations, leading to heterogeneity in the state of charge (Jo *et al.*, 2024*b* ▸; Gent *et al.*, 2016 ▸; Chung *et al.*, 2024 ▸; Lim *et al.*, 2025 ▸).

**Figure 5:**
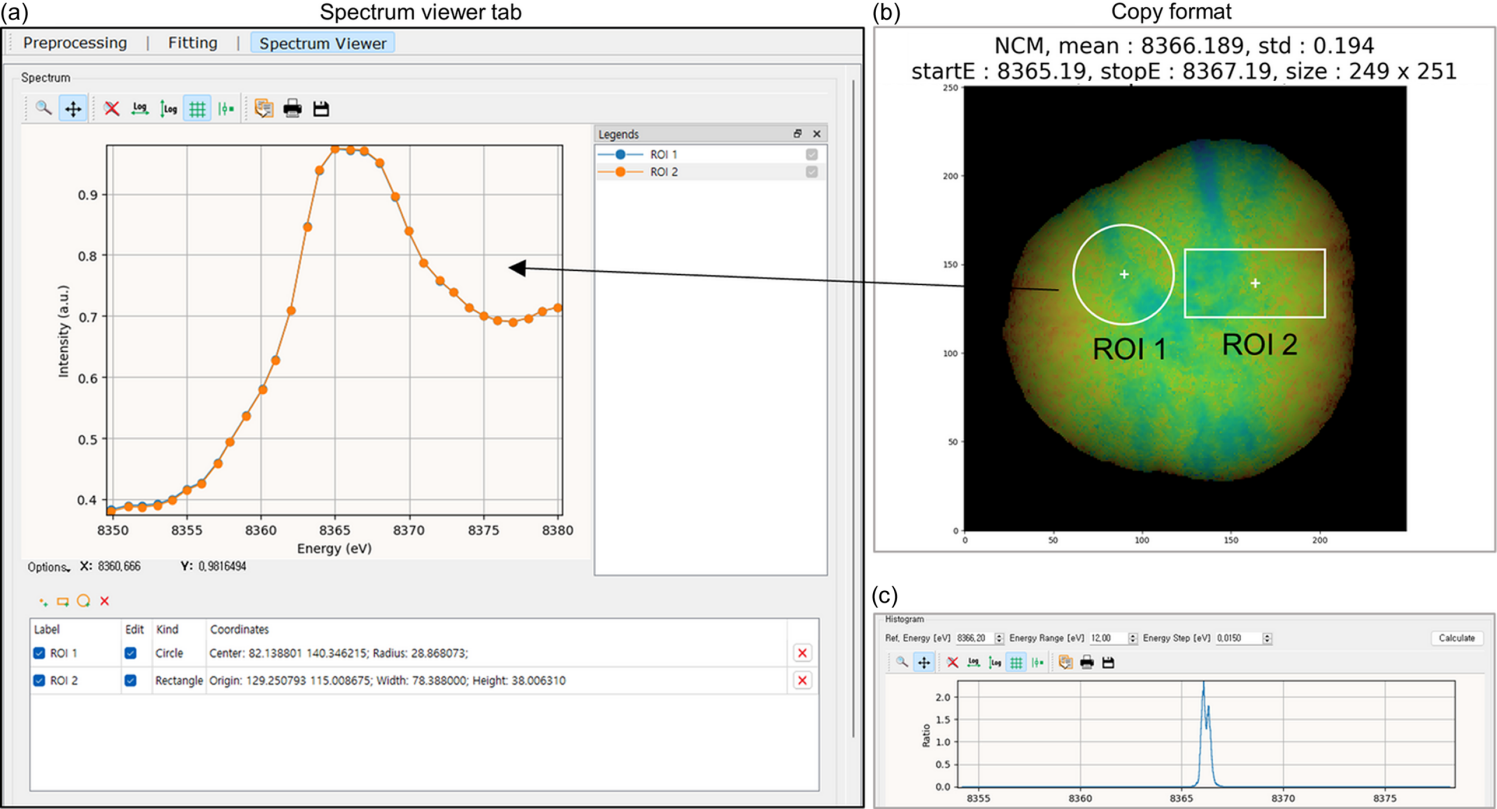
Screenshots of the spectrum viewer. (*a*) This spectral viewing can be used to verify the reliability of fitting results from specific pixels or ROIs. (*b*) Mean peak position energy, standard deviation, StartE, StopE and pixel dimensions are displayed above the color-mapped image. (*c*) Users can generate a histogram by setting values using ‘Ref. Energy’, ‘Energy Range’ and ‘Energy Step’.

Spectrum viewing can be conducted in the spectrum viewer tab [Fig. 5 ▸(*a*)]. In the display of color-mapped image, the mean of the peak position energy, standard deviation, StartE, StopE and pixel dimensions are shown above the image and can be easily copied by clicking the copy button [Fig. 5 ▸(*b*)]. In the histogram section, users can generate a histogram by setting the ‘Ref. Energy’ to establish the central energy value of histogram, define the *x* axis range in electronvolts using the ‘Energy Range’ and adjust the histogram width with the ‘Energy Step’ [Fig. 5 ▸(*c*)].

## 3. Software description

The *TXM-Pal* program was built in Python using libraries including *NumPy*, *SciPy*, *SILX* and *H5py*. For CPU-intensive tasks, *Rust* is integrated to manage CPU resources efficiently. This is complemented by the *Rayon* package, which optimizes multicore CPU utilization (Perkel, 2020 ▸) and is particularly advantageous for processing large image datasets. The *Rust*-implemented code includes functions for phase-cross-correlation, quadratic and Gaussian fitting, smoothing (Savgol, median, 3-point and boxcar), and renormalizing the energy scales in XANES stacks. With these capabilities, the program can analyze approximately 10 GB of XANES data within minutes on resource-constrained devices such as laptops. The user interface, developed using the *SILX* library, supports functionalities for imaging, spectrum analysis and masking (Vincent *et al.*, 2024 ▸).

**Figure 6:**
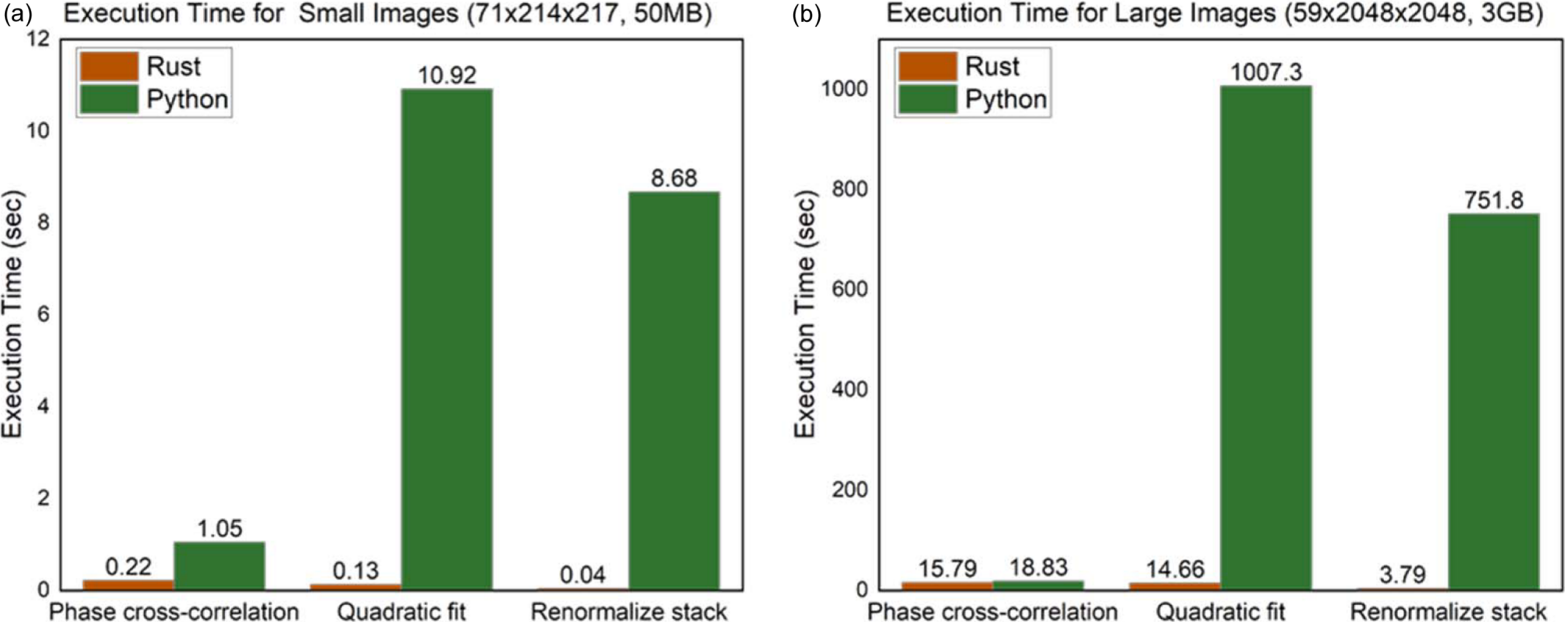
Comparison of execution time between *Rust* and Python for (*a*) small and (*b*) large images across tests for phase cross-correlation, quadratic fit and renormalize stack.

The *Rust*-based implementation in *TXM-Pal* offers data analysis speeds of up to tens of times faster than programs written only in Python. To demonstrate the performance benefits of the *Rust*-based implementation, tests were conducted on an Intel i7 7700HQ CPU at a base speed of 2.80 GHz, encompassing both small [71 × 214 × 217, Fig. 6 ▸(*a*)] and large [59 × 2048 × 2048, Fig. 6 ▸(*b*)] images. For the phase-cross-correlation algorithm, there was no significant difference in performance because *SciPy* library is well optimized for this task [‘Phase cross-correlation’ in Figs. 6 ▸(*a*) and 6 ▸(*b*)]. However, substantial differences in performance were observed for tasks such as quadratic fit and renormalization. Specifically, for large images (approximately 3 GB of XANES data), the *Rust*-based implementation in *TXM-Pal* exhibited data analysis speeds approximately 198× (3.79 versus 751.8 s) and 68× (14.66 versus 1007.3 s) faster than those possible with pure Python for the renormalize stack and performing quadratic fit, respectively [Fig. 6 ▸(*b*)]. These results highlight the significant advantages of implementing such operations in *Rust* rather than pure Python.

## 4. Summary and future development

We developed *TXM-Pal* as a software specially optimized for rapid and efficient XANES imaging data analysis at the 7C-XNI beamline of PLS-II. This software features a user-friendly GUI that significantly enhances data evaluation efficiency. Using *TXM-Pal*, we gained detailed insights into each step of the workflow, focusing on lithium-ion battery cathodes and mapping the Ni oxidation heterogeneity within cathode particles. For approximately 3 GB of XANES data, the *Rust*-based implementation in *TXM-Pal* achieved data analysis speeds approximately 198× and 68× faster than those possible with pure Python for the large-area renormalized stack and quadratic fit, respectively. Typically, the software enables XANES images to be processed within a few minutes. We plan to continuously update *TXM-Pal* to fully meet the needs of researchers at spectroscopic X-ray microscope beamlines. Future updates will include edge fitting and principal component analysis fitting via normalization.

## Data Availability

The software is freely available and can be accessed at https://github.com/physwkim/TXM-Pal/releases/latest.
